# Mr. Dunning, on the Jennerian Medal

**Published:** 1801-02

**Authors:** R. Dunning

**Affiliations:** Plymouth Dock


### To Dr. BATTY.

DEAR SIR,

THE active and unremitting exertions of the Editors of the Medical Journal to diffuse a general knowledge of the great advantages which result from the Jennerian Inoculation will, I have no doubt, ensure insertion in your next Number to the enclosed additional Testimonial, signed by nearly all the physicians and surgeons in these large towns. You will be much gratified to hear, that the ingenious physician of the fleet, Dr. Trotter, (than whom, I believe, there is hardly a man more alive to the extension of medical improvements) and the navy surgeons, are procuring an elegant and very appropriate GOLD MEDAL, to be presented to Dr. Jenner; an honourable and generous resolution, expressive at once of their great humanity, of their zeal for the service, and of their respect for, and attachment to science! Indeed it is very true, and I feel great pleasure in making the remark, that on this great occasion, as well as in the profecution of many other late interefting inquiries, thofe gentlemen have evinced an ardour of purfuit, which certainly reflects on them great reputation, and will, it may be hoped, bye and bye, (when it fhall be recollected how expenfive their claffical education, in the greater number of inftances muft neceffarily be, how much further expence, and how many years of indifpenfable and folicitous application, the nature of their fubfequent ftudies always requires) fecure to this truly refpectable and important clafs of officers, a larger fhare of public remuneration. I am, &c.

*Plymouth Dock, Jan.* 10, 1801. R. DUNNING.

